# A preliminary framework for managing sleep inertia in occupational settings

**DOI:** 10.1093/sleepadvances/zpad050

**Published:** 2023-11-18

**Authors:** Katya Kovac, Grace E Vincent, Jessica L Paterson, Cassie J Hilditch, Sally A Ferguson

**Affiliations:** School of Health, Medical and Applied Sciences, Appleton Institute, Central Queensland University, Wayville, SA, Australia; School of Health, Medical and Applied Sciences, Appleton Institute, Central Queensland University, Wayville, SA, Australia; Flinders University Institute of Mental Health and Wellbeing, College of Education, Psychology and Social Work, Flinders University, Bedford Park, SA, Australia; Fatigue Countermeasures Laboratory, San José State University, San José, CA, USA; School of Health, Medical and Applied Sciences, Appleton Institute, Central Queensland University, Wayville, SA, Australia

**Keywords:** shift work, sleep–wake cognition, sleepiness

## Abstract

Sleep inertia, the temporary period of impairment experienced upon waking, is a safety hazard that has been implicated in serious work-related incidents resulting in injuries as well as the loss of life and assets. As such, sleep inertia warrants formal management in industries where personnel are required to undertake their role soon after waking (e.g. emergency services, engineers, and health care). At present, there is a lack of practical, evidence-based guidance on how sleep inertia could be formally managed at an organizational level. We propose a preliminary framework for managing sleep inertia based on the translation of research findings into specific work procedure modifications/control mechanisms. Within the framework, work procedure modifications/control mechanisms to manage sleep inertia are organized into three levels: (1) modifications/controls that eliminate the chance of sleep inertia, (2) modifications/controls that reduce sleep inertia severity, and (3) modifications/controls that manage the risk of errors during sleep inertia. Practical considerations, limitations, and areas of further research are highlighted for each modification/control to help determine how readily each control measure could be implemented by industries at present. A guide for organizations to use this preliminary framework of sleep inertia management is put forward, as well as the next research priorities to strengthen the utility and evidence base of the framework.

This paper is part of the Sleep and Circadian Rhythms: Management of Fatigue in Occupational Settings Collection.

**Statement of Significance**

Sleep inertia, the temporary impairment experienced upon waking, is an occupational safety hazard for personnel in several safety-­critical industries. Studies suggest that sleep inertia is not being widely managed in a formal capacity, potentially due to a lack of formal guidance in sleep inertia risk mitigation. This review puts forth a novel sleep inertia management framework based on a synthesis of findings from field-based and experimental studies on sleep inertia. The sleep inertia management framework outlines specific work procedure modifications/control mechanisms to mitigate sleep inertia-related risk which can be operationalized by workplaces, with accompanying guidance on potential considerations and limitations. Finally, a research agenda is proposed to address the limitations of the control mechanisms identified in the sleep inertia framework.

## Purpose and Introduction

An occupational hazard is defined as an aspect of an occupation (e.g. the work role or work conditions) that has the potential to cause harm [1]. Occupational injuries as a result of workplace accidents are the fourth most common cause of work-related deaths, behind work-related cancer, circulatory diseases, and communicable diseases [2]. As such it is crucial to identify and manage occupational hazards to minimize the risk of harm in the workplace. An example of a well-established occupational hazard is fatigue. Work-related fatigue is defined as a state of impaired mental or physical performance capability due to sleep loss or extended wakefulness, circadian phase, or workload that results in the reduced capacity to safely perform duties [3]. It is well known that the presence of fatigue can increase the likelihood of workplace errors leading to accidents, injuries, or deaths [4]. Fatigue management policies and procedures are used in industries where fatigue-related safety risks are high to reduce the risk of harm due to fatigue-induced errors or incidents [4, 5]. Another hazard that should be taken into account when thinking about fatigue-related risk is sleep inertia.

### Sleep inertia

Sleep inertia is the unavoidable post-waking period during which cognitive and physical performance, as well as alertness, are temporarily degraded as an individual transitions from sleep to wake [6]. It is commonly experienced as feelings of grogginess or disorientation. Importantly, the impairments associated with sleep inertia impact effective and safe work performance due to the effects on decision-making, problem-solving, reaction time, driving ability, and physical abilities such as balance and coordination [7–11]. Impairments associated with sleep inertia are most severe immediately upon waking and gradually dissipate as time awake progresses. However, the time course of sleep inertia is equivocal with some studies finding a return to baseline performance within minutes and others finding that effects can last up to 2 hours post-waking [12–14]. In addition, the severity of performance impairments can be influenced by several sleep- and circadian-related factors. These include timing of waking, particularly during the circadian trough (typically between 0300 and 0500 for those with a regular sleep period of 2300–0700) [8, 15], waking during deep sleep (i.e. slow wave sleep [SWS]) [8, 16], and waking after prior sleep loss related to acute or chronic sleep deprivation or chronic or acute sleep restriction [8, 16–18].

Decades of research investigating the outcomes of sleep inertia under experimental conditions have demonstrated the objective risk that sleep inertia poses for individuals performing important or safety-critical tasks soon after waking [6, 19]. In the occupational setting, this is particularly relevant for individuals with on-call arrangements. On-call arrangements are used in several high-risk industries such as emergency services, healthcare, aviation, utilities, and engineering to provide 24/7 service coverage for emergency or urgent situations [20, 21]. Given that on-call shifts are typically scheduled during non-peak times, such as during the night, emergency callouts can occur when on-call personnel are sleeping. When called, personnel are typically required to respond quickly to calls and thus can be driving and navigating to an emergency site, making critical decisions for themselves or their teams, problem-solving, or performing their work role while experiencing sleep inertia [22]. Individuals who are experiencing sleep inertia while performing safety-critical tasks are at greater risk of performance errors or accidents, putting themselves and those around them at risk of harm.

Beyond on-call personnel, shift workers who are given the opportunity to nap can also be impacted by sleep inertia if their nap periods are interrupted by emergency incidents requiring their response. In particular, shift workers with extended work shifts (scheduled 10–12-hour shifts or unscheduled long shifts) may be allowed to nap on shift [23]. Extended shifts are commonly used in industries such as the military, emergency services (fire and rescue, ambulance services), and healthcare [24, 25]. Significantly, shift-workers with extended shifts are at risk of severe sleep inertia given that they are often experiencing chronic sleep debt [24, 25].

### Consequences and outcomes of sleep inertia in the workplace

The safety risk that sleep inertia poses is significant and the phenomenon has been implicated in several serious transportation accidents resulting in the loss of life and assets [26–29]. For example, 158 civilians died in a plane accident after the Captain took control after a cockpit nap and failed to follow the standard operating procedure for controlled rest which considers length of nap and timing of the nap relative to time of day [28]. Beyond such large-scale incidents, several qualitative investigations have revealed the impacts and outcomes of sleep inertia on personnel in the field [22, 30–35]. Firefighters identified sleep inertia as a factor contributing to navigation errors at an incident site resulting in delays in arrival [33]. In addition, ambulance personnel attributed sleep inertia to an incident where the wrong team was deployed to an incident in a significantly different geographical location [34]. Furthermore, emergency personnel (volunteer and salaried firefighters and ambulance personnel) have indicated concerns about sleep inertia impacting their performance [31, 34]. Specifically, emergency service personnel reported concerns about their ability to safely drive to incident sites and their ability to perform their work role effectively while experiencing sleep inertia [34]. Personnel have also expressed concerns about the subsequent emotional, mental, and financial impacts that sleep inertia-related incidents may have for them, should they eventuate [34].

### The current state of sleep inertia management

There have been few studies investigating how sleep inertia is being managed in the field, with the existing studies being primarily conducted with emergency service personnel. In these studies, personnel have reported several informal strategies used to personally manage sleep inertia experienced as part of their role [30, 33–35]. For example, firefighters have reported to manage their expected grogginess by preparing their clothes and arranging their important belongings (phone, wallet, and keys) in easy-to-reach places to facilitate their response to a call-out [33]. Other personnel reported using strategies to reduce perceived sleep inertia, such as winding down the windows to induce cold temperature shock and running to and from their car to wake themselves up [33]. United States Coast Guard pilots have reported to manage sleep inertia while on duty by ingesting caffeine, splashing water or using a damp cloth on their face, and white light exposure [30]. It is important to note that of the informal strategies reported by personnel, to date, only caffeine ingestion prior to a nap and light exposure upon waking have been found to objectively reduce sleep inertia [36, 37]. Other informal strategies such as physical movement/exercise and cold temperature shock do not currently have a strong evidence base for reducing sleep inertia [38]. In addition, although some personnel reported informal strategies to manage sleep inertia, others did not have any knowledge or strategies on how to manage this potential hazard.

Given the risk that sleep inertia poses for the safety and performance of personnel in several industries, it is important that sleep inertia, like any other workplace hazard, is appropriately and systematically managed. Despite this, the same studies on sleep inertia with personnel from emergency service industries found that sleep inertia is not widely managed in a formal capacity within workplaces [34]. While not explored in previous studies, the reasons for the lack of formal management of sleep inertia may be due to the lack of clear guidance on what can be done to manage sleep inertia. Thus far, empirical studies have examined various sleep inertia countermeasures (i.e. strategies to reduce sleep inertia such as caffeine ingestion [36, 39] and light exposure [37, 40]); however, there is no guidance about how these countermeasures can be practically implemented into the workplace. Rather, a recent study exploring the practicality of sleep inertia countermeasures as perceived by emergency service personnel identified several practical barriers to the implementation of current evidence-based countermeasures (e.g. a lack of time to enact the strategy) [34]. Furthermore, eliminating sleep inertia is not the only way to manage sleep inertia as a hazard. Dawson, Ferguson, and Vincent [41] highlighted the possibility of re-­proceduralizing tasks to ensure workers perform tasks safely while experiencing sleep inertia in situations where eliminating sleep inertia is not possible. Ideally, empirical studies would also be conducted to determine the effectiveness of management strategies in decreasing the risk of sleep inertia to support the motivation for industries to implement these strategies. To date, this current review of the literature offered few empirical studies which have tested the effectiveness of sleep inertia management strategies in reducing the risk of errors and accidents in the field [42].

It is clear that sleep inertia is an occupational hazard that merits formal management in the workplace to reduce the risk of potential harm to personnel. The handful of exploratory studies on sleep inertia conducted with workers suggests that it is not being widely managed which is unsurprising given the lack of clear and systematic published guidance on sleep inertia risk mitigation. Based on these gaps in the literature, we propose a preliminary framework on how to manage sleep inertia which could be used by workplaces. To achieve this, the existing literature on field-based and experimental studies on sleep inertia was synthesized and findings translated into specific strategies that could be operationalized. It should be noted that the majority of the existing studies on sleep inertia management have been conducted with emergency service personnel and so within the proposed framework there is frequent reference to examples from the emergency service industry. We believe, however, that the framework proposed here can be applied to occupations beyond emergency services where sleep inertia is a problem and similar on-call and/or shift-working conditions apply. Following this framework, a research agenda is proposed to target the gaps in our understanding of how to best manage sleep inertia and to develop the utility and evidence base for the effectiveness of sleep inertia management strategies.

## A Preliminary Framework for How Sleep Inertia Could be Managed

Workplace hazards are broadly managed by occupational and safety management systems; clearly outlined guiding procedures and strategies that a workplace implements to reduce the risk of harm [43]. It is important to note that hazards do not always result in harm, rather exposure to a hazard can increase the risk or likelihood that harm could occur. Different types of hazards require different systems of management. For example, Fatigue-Risk Management Systems (FRMS) are typically used in high-­fatigue-risk industries. FRMS are defined as “a data driven set of management practices for identifying and managing fatigue-­related safety risks” [44]. FRMS can involve procedures, strategies, or rules that reduce the risk of fatigue occurring in the first place [41]. Sometimes fatigue is unavoidable and so strategies need to be put in place to reduce the likelihood of harm when exposed to fatigue [41, 45]. Sleep inertia could be managed within a FRMS; however, clear guidance regarding the relevant control mechanisms is needed.

**Figure 1.**
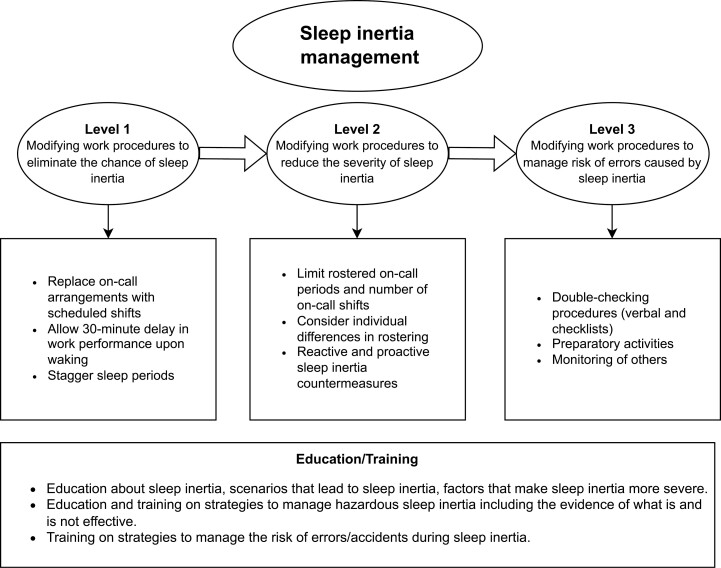
A preliminary sleep inertia framework. The sleep inertia management framework illustrated outlines work procedure modifications related to eliminating sleep inertia (level 1), reducing the severity of sleep inertia (level 2), managing the risk of errors caused by sleep inertia (level 3), and education and training on sleep inertia and management strategies.

The content of the current sleep inertia management framework described in this review was guided by existing fatigue-risk management frameworks, particularly the fatigue-risk trajectory model put forth by Dawson and McCulloch [5]. In brief, Dawson and McCulloch [5] describe several levels of potential fatigue-­related hazards and appropriate risk management controls at each level of the fatigue-risk trajectory. At the top levels (levels 1–2), controls for fatigue hazards are related to fatigue reduction (e.g. ensuring sufficient sleep). At the lower level (level 3), controls are related to facilitating safer performance despite the experience of fatigue, a strategy labeled by the authors as “fatigue-proofing” [45]. The proposed sleep inertia framework (Figure 1) follows a similar structure that begins with controls to eliminate the chance of sleep inertia (level 1). If it is not possible to eliminate sleep inertia, the subsequent controls relate to reducing the severity of sleep inertia (level 2), and sleep inertia proofing to manage the risk of errors/accidents as a result of sleep inertia (level 3). Given that the control mechanisms outlined in this framework focus on changing work procedures to manage sleep inertia, we have made this explicit in the level heading titles e.g. “Level 1: Modifying work procedures to eliminate the chance of sleep inertia.” However, to remain consistent with fatigue-risk management terminology, we will still be referring to these work procedure modifications as “control mechanisms” throughout. Each control mechanism is further divided into categories including: personnel (control mechanisms requiring actions or behaviors to be undertaken by personnel), management (control mechanisms requiring input from management), operations (control mechanisms requiring changes to operating procedures), schedules (control mechanisms requiring changes to scheduling), and training (control mechanisms requiring changes to personnel training) to provide guidance as to what area of the organization might be responsible for managing and/or implementing the control mechanism (Table 1).

**Table 1.** A Summary of Work Procedure Modifications/Control Mechanisms to Manage Sleep Inertia in the Sleep Inertia Management Framework

Level	Category	Work procedure modification/ control mechanism	How it manages sleep inertia	Considerations and/or limitations of control mechanism	Applicable further research
1	Scheduling	Remove on-call periods^b^	• Eliminate sleep inertia by removing unpredictable wake times.	• Financial impact to industries to provide 24/7 active coverage	
1	Operations	A 30-minute delay in work performance upon waking^b^	• Eliminate the influence of sleep inertia on work performance by allowing time for sleep inertia to dissipate before engaging in safety-critical tasks.	• Delays in response times are not always possible for emergency incidents.	
1	Operations	A 30-minute delay in work performance upon waking for only non-urgent incidents^c^	• Eliminate the influence of sleep inertia on work performance due to allowing time for sleep inertia to dissipate.	• Formalize what constitutes an urgent versus a non-urgent incident.	
1	Operations	Stagger sleep/nap periods so that there is always one person awake to take on critical tasks^c^	• Eliminate the influence of sleep inertia on workplace performance for personnel who are undertaking critical tasks.	• Potential impact of fatigue on the person that remains awake if they are required to respond prior to having their nap period.	
2	Scheduling	Improve shift scheduling (including reduced on-call shifts) to minimize sleep loss^b^	• Reduce severity of sleep inertia by reducing sleep loss. • Reducing the number of on-call shifts also reduces the amount of nighttime wakings and therefore exposure to sleep inertia.	• Logistics and financial considerations around restructuring scheduling and staffing requirements.	
2	Scheduling	Schedule shifts according to individual differences in sleep inertia susceptibility^a^	• Tailor shifts to avoid high sleep inertia risk, based on known individual differences in timing and severity.	• Must be able to determine an individuals’ susceptibility to sleep inertia—more research is needed.	• More research on sleep inertia and individual differences in general is needed.
2	Operations/ Personnel	Implement proactive sleep inertia countermeasures such as caffeine ingestion prior to a nap for predictable on-shift naps^c^	• Reduces severity of sleep inertia upon waking from on-shift naps	• Requires predictable wake times. • Identify potential motivational barriers (e.g. some individuals may not like using caffeine). • Potential impact of proactive sleep inertia countermeasures on subsequent sleep.	• Given potential motivational barriers to caffeine ingestion, more research is needed into alternative proactive sleep inertia countermeasures.
2	Operations	Implement the use of reactive sleep inertia countermeasures^a^	Reduces severity of sleep inertia during response to calls	• Equipment needed (e.g. affordability, physical implementation). • How to implement the countermeasure without critically delaying response times. • Identify potential motivational barriers to the uptake of this countermeasure. • Potential impact of reactive sleep inertia countermeasures on subsequent sleep.	• Field-based research investigating how to practically implement countermeasures including identifying and addressing issues such as management/personnel buy-in (motivation to use) and assessing feasibility. • Development of effective and affordable equipment to administer reactive sleep inertia countermeasures.
3	Operations/ Management/ Personnel	Pre-sleep preparation for tasks required immediately upon wake-up^d^	• Reduce likelihood of sleep inertia-related errors by reducing cognitive load of tasks upon waking.	• Education and training of personnel on what preparatory strategies/tasks they could undertake to reduce cognitive load upon waking. • The applicable strategies may need to be tailored to the workplace and type of work.	
3	Operations/ Management/ Personnel	Monitoring self and team members for sleep inertia and reassigning tasks^d^	• Reduce likelihood of sleep inertia errors by identifying personnel who are experiencing sleep inertia and reassigning critical tasks accordingly.	• Education and training of personnel on what factors to be aware of (e.g. slowed responses, lack of communication and engagement) and when it might be more important to be vigilant (e.g. during nighttime callouts). Support the monitoring and identification of individuals who are experiencing severe sleep inertia for the purpose of safety, so as not to discourage people from reporting others or themselves.	
3	Operations/ Management/ Personnel	Undertaking double-checking procedures (verbally and using checklists) of critical tasks^d^	• Reduce the likelihood of errors associated with sleep inertia by ensuring critical tasks are checked.	• Training on how and when to undertake double-checking procedures. • Embedding double-checking processes into standard procedures. • Devise checklists encompassing the critical tasks that typically occur upon waking to an emergency response.	
Education	Training	Education on sleep inertia^d^	• Increase personnel awareness of what sleep inertia is, how it can impact them, and when they are most likely to be impacted.	• Refer to existing evidence base on sleep inertia.	
Education	Training	Education on evidence-based strategies to manage sleep inertia and strategies with limited evidence base^d^	• Reduce personnel reliance on strategies that have limited evidence base.	• Refer to existing evidence base on sleep inertia countermeasures.	
Education	Training	Training in how to implement and undertake evidence-based strategies to reduce sleep inertia (e.g. sleep inertia countermeasures) or manage sleep inertia^a^	• Reduce likelihood of sleep inertia-related errors by providing personnel with the skills to reduce sleep inertia severity or manage sleep inertia deficits.		More research is needed prior to implementing this.

**Caption:** A summary of the different work procedure modifications/control mechanisms to manage sleep inertia, how the work procedure modification/control mechanism manages sleep inertia, and considerations and limitations of the work procedure modification/control mechanism organized by level and category and the current feasibility of implementing each work procedure modification/control mechanism (in superscript).

Superscript letters for each work procedure modification/control mechanism indicates the current feasibility of implementing each work procedure modification/control mechanism with considerations of further research needs and potential limitations.

^a^More research is needed;

^b^Work procedure modification/control mechanism could be implemented but potentially impractical;

^c^Work procedure modification/control mechanism could be implemented but some limitations exist or more research is needed;

^d^Work procedure modification/control mechanisms could be implemented with few or no limitations.

### Level 1: Modifying work procedures to eliminate the chance of sleep inertia

Level 1 of the proposed sleep inertia management framework involves modifying work procedures to eliminate the potential for sleep inertia and, therefore, the possibility of sleep inertia-­related errors. The most obvious control would be to simply replace all on-call shifts with a predictable set shift. Removing on-call arrangements would significantly limit the possibility that personnel will be awoken to respond to an emergency incident, thus removing the hazard of sleep inertia completely. While this safety measure could eliminate sleep inertia risks related to on-call work, there are some significant limitations. First, shift work arrangements would need to replace on-call shifts to provide a 24/7 service, and the use of such arrangements may not be viable for certain industries. For example, for firefighters, emergency incidents can be sporadic depending on the season and geographic location, e.g. rural or metropolitan [46]. In addition to the sporadic nature of incidents, emergency volunteers (e.g. firefighters, ambulance, and state emergency services) with on-call arrangements also often hold daytime jobs. As a result, providing active 24/7 coverage during non-peak times, such as during the night, is not feasible and could lead to other work health and safety hazards, such as circadian misalignment and sleep loss. The potential for eliminating sleep inertia by removing on-call arrangements would be, for the most part, unworkable for several industries.

For on-call, shift napping and extended operation arrangements, eliminating sleep inertia for personnel could also be achieved by implementing a 30-minute protection window where personnel are prevented from undertaking any work within the first 30 minutes post-waking. Sleep inertia typically dissipates within 30 minutes of waking and so delaying all tasks for 30 minutes post-waking could eliminate the influence of sleep inertia on tasks completed after the 30-minute delay [47–49]. Implementing a delay of 30 minutes for all tasks may not be viable where a delay in response to emergency incidents can be detrimental. A potential middle ground could be delaying response times for non-urgent incidents. According to firefighters, an example of an urgent incident is a motor vehicle accident, whereas a fallen tree obstructing a road, while still dangerous, is not considered an incident that would require an immediate response, so as to mitigate the risk of sleep inertia [33]. Given that sleep inertia dissipates over time post-waking, delaying response times for non-urgent incidents could reduce the likelihood that personnel will be driving to emergency sites or attending incidents while experiencing severe sleep inertia. Delaying response times for non-urgent incidents may be effective given that firefighters have reported that sleep inertia can be worse during non-urgent incidents due to a lack of an adrenaline response [33]. In implementing a delay for non-urgent incidents, it would be important for workplaces to clearly define what constitutes an urgent and non-urgent incident so that personnel know when they can delay their performance in order to recover from sleep inertia.

Another strategy to eliminate the impact of sleep inertia on those undertaking critical tasks may be to stagger sleep opportunities. By staggering sleep opportunities, this ensures that there is always someone available to respond to emergency incidents who can be assigned responsibility for critical tasks. Given that the person who is assigned the most critical task will already be awake, they will not be impacted by sleep inertia. For example, in a firefighting scenario, the person who is awake will be the designated driver (critical task) for their wake period and the remaining personnel who are awoken will ride along preparing for the incident (non-critical task) while allowing for sleep inertia to dissipate during the ride to the incident site. A potential limitation of this strategy is that for overnight shifts, the individual who remains awake may also experience performance decrements as a result of sleep deprivation due to remaining awake, and/or sleepiness due to being awake when they would normally be asleep.

### Level 2: Modifying work procedures to reduce the severity of sleep inertia

Level 2 of the sleep inertia management framework involves modifying work procedures to reduce the likelihood of severe sleep inertia, which could lead to performance errors or accidents made by personnel. Given that sleep loss exacerbates sleep inertia, organizing schedules in a way that improves sleep and minimizes sleep loss is an important control [8, 17, 18]. On-call arrangements are associated with sleep restriction, even when no calls are received and so limiting the number of on-call shifts and time on-call is a key control [50, 51]. One way to manage sleep inertia in the field includes rostering volunteers who are normally on-call 24/7 every day of the year in a different pattern, namely on for blocks (e.g. 2 weeks) and then off for blocks [34]. This would mean that volunteers who are normally always on-call would have some protected time between on-call periods. For personnel that have set on-call periods, scheduling changes could include reducing the period of the on-call shift to minimize potential sleep loss associated with being on-call. Overall, the less time that individuals are on-call, the less time they are potentially exposed to sleep inertia and the associated risks. As such, minimizing on-call periods serves to both reduce the potential severity of sleep inertia and the likelihood that personnel will be impacted by sleep inertia.

Another schedule-related change that may reduce the likelihood of severe sleep inertia is the consideration of individual differences in chronotype or vulnerability to sleep inertia in scheduling. Individual differences may play a role in the experience and vulnerability to sleep inertia; however, more research is needed in this space. For example, some personnel report feeling alert almost immediately after waking, while for others, optimum alertness is delayed for several minutes post-waking [33]. Other recent studies have also found significant interindividual differences in the subjective experience of sleep inertia [52, 53]. Furthermore, subjective ratings of performance during sleep inertia do not always match objective performance indicating poor self-assessment of performance during the sleep inertia period [54]. More studies are needed to determine whether subjective measures of sleep inertia match objective measures such that individuals who feel like they do not experience sleep inertia, also do not perform worse after waking.

A further factor related to individual differences which could be considered in scheduling is chronotype [55]. Chronotype is an individual’s natural preference for either going to sleep late or waking late (“evening” or “late” type), or going to sleep early and waking early (“morning” or “early” type) [56]. Preliminary studies have found that an individual’s chronotype may affect sleep inertia, with late chronotypes experiencing worse sleep inertia during habitual wake times compared to early chronotypes [55]. More studies are also needed to determine whether strategically scheduling on-call shifts and on-shift nap times using an individual’s chronotype is effective in reducing the severity of sleep inertia and the incidences of sleep inertia-related accidents. If effective, several logistical caveats may need to be considered such as the potential for emergencies or a high workload preventing napping at scheduled times, and whether there is enough staff variation in chronotype to separately schedule people according to their chronotype [23]. In addition, while scheduling in this way may suit the chronobiology of individuals, it may not always align with the individual’s other occupational, family, or domestic responsibilities, and so these factors must also be considered to avoid unintended consequences elsewhere. Overall, however, it is important to note that at present we know very little about the individual differences in vulnerability to sleep inertia. Future research is therefore first needed to determine and better understand the individual determinants of sleep inertia vulnerability.

Managing the potential for severe sleep inertia could also involve the use of proactive and reactive sleep inertia countermeasures. Proactive sleep inertia countermeasures are countermeasures that are implemented prior to sleep [57]. For example, caffeine administered prior to a brief nap has been demonstrated as effective in reducing sleep inertia upon waking [36, 58]. This safety measure would be useful to manage the severity of sleep inertia after on-shift naps, given that wake times from on-shift naps can be planned. However, this strategy is not useful when wake times are not predictable such as in emergency response scenarios. The unpredictable nature of on-call shifts can preclude the use of proactive sleep inertia countermeasures for several industries and so reactive sleep inertia countermeasures may be more useful.

A reactive sleep inertia countermeasure is one that can be used after waking. While no reactive sleep inertia countermeasure is reported to eliminate sleep inertia, a targeted amount of light administered upon and after waking using specialized equipment (goggles and masks) has been demonstrated as effective in reducing sleep inertia severity [37, 40]. However, despite the efficacy of these reactive sleep inertia countermeasures, there are limitations to their implementation in the workplace. For example, only one of these countermeasures has undergone field testing. Hilditch et al. [42] highlighted the challenges of translating laboratory-validated countermeasures to real-world environments. In an at-home setting using a field-deployable light source, the study showed that while light was able to modestly improve alertness, mood, and working memory after nighttime awakenings from deep sleep, performance on a visual task was worsened in the light condition. This may have been due to visual interference from the light source itself, highlighting the importance of translational studies to test not only the effectiveness of a specific device, but also the implementation for specific operational tasks and environments. Another potential limitation of both proactive and reactive sleep inertia countermeasures is the effect of sleep inertia countermeasures on subsequent sleep [59]. This may be particularly pertinent after short callouts where personnel will have the opportunity to sleep after returning from a call-out or following false alarms. If sleep inertia countermeasures prevent workers from being able to return to sleep, then this can lead to sleep loss which could unnecessarily affect next-day safety and performance [59]. As such more research is needed to determine the effects of sleep inertia countermeasures on subsequent sleep [59].

In previous studies, personnel have reported that time taken to employ sleep inertia countermeasures and time for the sleep inertia countermeasure to take effect are the primary limiting factors to sleep inertia countermeasure use [34]. As such, studies must be conducted to determine how effective sleep inertia countermeasures could be integrated into response procedures without delaying response times. Firefighters report informally integrating strategies to manage sleep inertia as part of their response to emergency callouts, for example, running to their car to help wake up or winding down windows for the alerting effect of the cold temperature [33]. These examples demonstrate the feasibility of integrating potential sleep inertia countermeasures into response procedures without delaying response times, though efficacy has not been confirmed. For example, Reyner and Horne [60] found that exposure to cold air and listening to the radio was only temporarily effective in reducing subjective sleepiness during driving. Other studies suggest that there may also be several motivational barriers to using sleep inertia countermeasures, such as concerns regarding comfort in the case of exercising, exposure to bright light, and chewing caffeinated gum upon waking [34]. Both practical and motivational barriers to sleep inertia countermeasures will need to be addressed during their development and implementation in order to facilitate uptake in the field. It should be noted that once effective and practical proactive and reactive sleep inertia countermeasures are determined, organizations that choose to incorporate these countermeasures will need to provide the appropriate training and equipment for personnel to implement these countermeasures (discussed further in *Education on sleep inertia and strategies to manage sleep inertia*).

### Level 3: Modifying work procedures to manage the risk of errors/accidents caused by sleep inertia (sleep inertia proofing)

Finally, level 3 of the sleep inertia management framework involves managing the risk of errors/accidents when sleep inertia is unavoidable. Primarily, this could be achieved by implementing strategies that facilitate safe performance while experiencing sleep inertia. Dawson, Chapman, and Thomas [45] identified several “fatigue proofing strategies” used by personnel in high-risk industries that facilitated safe performance while fatigued. These strategies could be adapted to be sleep inertia-­proofing strategies. Error-proofing strategies are often informal and specific to the context of the industry [33, 35, 45]. Sleep inertia-­proofing strategies identified in previous studies with firefighters included informal preparatory activities, such as laying out clothes before going to bed to facilitate a quick response to callouts and to reduce the chance of forgetting something [33]. Firefighters in the same study also reported monitoring the safety of teammates during night-calls, an informal behavior that may be facilitated by the culture of mate-ship and camaraderie involved in volunteer firefighting [33, 61]. Personnel also recommend the use of double-checking procedures [34]. Double-checking procedures may involve personnel checking their decisions or performance with another team member to reduce the likelihood of errors. Dawson, Chapman, and Thomas [45] reported on similar procedures used in the maritime industry, describing how marine pilots use hand signals and ask the captain of the boat to call back verbal navigation commands as a double-checking procedure. The use of checklists may be another type of double-checking procedure and one that is already effectively implemented as a tool for error management in the aviation industry [62]. As an error management tool, checklists include a list of actions or criteria that can aid in the memory, tracking, and standardization of critical tasks [62]. To help manage the likelihood of sleep inertia-related errors, a checklist could be devised and implemented specifically for the critical tasks or procedures that personnel typically undertake when responding to incidents upon waking.

Sleep inertia-proofing strategies will likely be industry-­specific, and so it is important that research is conducted to identify appropriate error-proofing strategies for each industry [45]. Once informal sleep inertia-proofing strategies are identified, their efficacy in reducing sleep inertia-related incidents should be tested. Efficacious sleep inertia-proofing strategies could then be disseminated amongst the relevant industries and formalized at the organizational level as part of workplace safety procedures to reduce the potential for sleep inertia-related errors or accidents and improve personnel safety.

### Education on sleep inertia and strategies to manage sleep inertia

To facilitate the implementation and uptake of the control mechanisms discussed in the sections above, further education on sleep inertia, as well as training on the different control mechanisms to manage sleep inertia, would be needed. Information and training related to sleep inertia could be integrated into existing fatigue management education resources and training sessions. Education and training on sleep inertia should include information about what sleep inertia is, the specific scenarios in which personnel may experience sleep inertia as part of their emergency service role, the factors which can exacerbate sleep inertia, and the potential risks to safety that sleep inertia can pose for emergency service personnel (e.g. the risk of errors and accidents). If personnel are aware that they may experience sleep inertia and are aware of the conditions that may result in severe sleep inertia, then they may operate with more caution when waking to respond to calls. Indeed, a systematic review of the effect of fatigue management training with emergency medical service personnel found that education on fatigue management, including information on fatigue and strategies to manage fatigue, resulted in improved safety behaviors and outcomes [63].

In addition to educating personnel about sleep inertia, it would be advantageous to provide personnel with strategies that they could use to manage sleep inertia, such as the control mechanisms described in levels 2 and 3 of the sleep inertia management framework. While the study by Kovac, Ferguson, Vincent, and Paterson [33] found that some firefighters already informally undertake strategies to manage the risks of sleep inertia, the sample in this study had an average of 9 years of experience and so it is possible that these strategies were developed with experience over several years. In comparison, new personnel may not have had the chance to develop strategies to manage sleep inertia. Indeed, previous research on the safety behaviors of construction workers found a significant decrease in the risk of work-related accidents with increased age, potentially due to the increased experience in the role and the development of safety behaviors [64]. As a result, new emergency service recruits could benefit from the formal organizational-level dissemination of strategies to manage sleep inertia and improve safety during the early stages of their career.

Once effective evidence-based strategies to manage sleep inertia are developed, these strategies could be included in education and training programs. Alongside this, it is important to educate personnel about the potentially limited efficacy of specific sleep inertia countermeasures that they may informally undertake to reduce or manage sleep inertia. For example, while personnel in previous qualitative studies have reported that they informally use exercise as a strategy to wake themselves up, research into exercise as a sleep inertia countermeasure found that a 30-second burst of exercise is not effective in reducing the cognitive aspects of sleep inertia, although a longer period may be effective [33, 34, 38]. Research has also shown that people are not necessarily good at judging their own sleep inertia impairment and so education should also include information on this misperception [54]. If personnel are aware that certain strategies are not effective at objectively reducing sleep inertia, and that they may misperceive their sleep inertia impairments, then this may encourage them to remain cautious of their performance while experiencing sleep inertia even when using strategies to assist in waking up.

## Future Directions and Research Agenda

Based on findings from quantitative laboratory-based studies and qualitative field studies on sleep inertia, we have proposed a preliminary framework for sleep inertia management. As identified in Table 1, there are controls that can be implemented by organizations in the short term, whereas others require further consideration.

## What Can Workplaces do Right Now?

Workplaces can use this preliminary sleep inertia management framework to identify specific control mechanisms that are applicable and practical for implementation in their work settings. Table 1 should be used as a guide for some of the considerations and limitations associated with each strategy and those that require further research prior to implementation. As identified in Table 1, some control mechanisms can be implemented with minimal further research. For example due to the significant evidence base on sleep inertia and the demonstrated benefit of education as a safety measure in improving safety performance [63], educating personnel about sleep inertia is one safety measure that could potentially be implemented by emergency service industries immediately. Furthermore, some of the error-proofing controls such as double-checking procedures during times where individuals are susceptible to sleep inertia could be implemented with minor changes to operations and education on how the control could be carried out. Given that the process of developing and testing effective and practical sleep inertia countermeasures will take time, it is important that organizations focus on the workplace procedures that they can modify to minimize the effects of sleep inertia, as outlined in this proposed sleep inertia management framework.

## What Requires Further Research?

### Sleep inertia countermeasures

Prior to the implementation of sleep inertia countermeasures, more field-based research is needed to determine how proactive and reactive sleep inertia countermeasures could be practically implemented in workplaces and their efficacy in specific operational environments. For example, in the case of caffeine ingestion prior to a nap, further research is needed to determine the best way of administering the caffeine (e.g. gum, tablet, and drink). Existing studies also suggest that there may be motivational barriers around the use of caffeine as a sleep inertia countermeasure, for example, if personnel are negatively affected by caffeine or oppose the use of artificial stimulants [34]. For light exposure upon waking, further research is needed to develop the most effective and practical equipment to administer the light to personnel without delaying response times or interfering with visual tasks. Equipment must also be affordable for workplaces given the number of personnel who may need to access the equipment. As such, ongoing research into other sleep inertia countermeasures is warranted to provide further options to personnel.

### Scheduling based on individual differences

Preliminary research suggests that individuals are impacted by sleep inertia to a different extent and this information could be considered during scheduling. Prior to this, further research is first needed to identify the traits or individual determinants that make an individual susceptible to experiencing objectively severe sleep inertia. Based on preliminary findings, the influence of chronotype on sleep inertia warrants further investigation, particularly the effects of chronotype on waking during non-habitual wake times. If traits related to sleep inertia susceptibility are identified, further research may be needed to develop objective or subjective measures of that particular trait to identify differences between individuals. Such measures could then be implemented as part of recruitment procedures to help inform scheduling based on susceptibility to sleep inertia. An important consideration here is that, to date, several studies have only demonstrated a subjective difference in sleep inertia severity between individuals, rather than an objective difference [52, 53]. Furthermore, research suggests that there tends to be a misalignment between an individual’s subjective perception of their sleep inertia impairment compared to their objective performance [54]. As such, there is a need for more research into the individual traits that influence objectively measured sleep inertia severity.

### Control mechanism tailoring, evaluation, and monitoring for improvement

As noted within the sleep inertia management framework, there is a need for individual workplaces to tailor sleep inertia control mechanisms to their own work setting. Future research could investigate risk-based interventions tailored to specific work settings. In addition, there is a need to determine the specific processes that workplaces must undertake to rigorously monitor and evaluate the effectiveness of control mechanisms in reducing sleep inertia-related safety risks, and as part of the continuous improvement model of safety management.

## Conclusion

A preliminary framework for sleep inertia management has been put forth by synthesizing and translating existing research findings into practical control mechanisms that could be implemented in occupational settings. The work procedure modifications/control mechanisms within this sleep inertia management framework target potential sleep inertia-related risk through three levels: level (1) eliminating the chance of sleep inertia, level (2) reducing the severity of sleep inertia, and level (3) managing the risk of errors during sleep inertia. For each modification/control measure, practical considerations, limitations, and future research needs have been identified. It is important to note that more research is needed to implement the framework as a whole. However, certain control measures, such as wider education on sleep inertia, and sleep inertia-proofing strategies such as double-checking procedures, could be broadly and readily implemented by industries immediately. It is recommended that industries, agencies, and workplaces impacted by sleep inertia consider this preliminary framework for sleep inertia management as a guide in tailoring control mechanisms to best suit their work setting. Furthermore, it is also recommended that sleep inertia control mechanisms are continuously evaluated and monitored for their effectiveness in reducing sleep inertia-related errors and incidents. In doing so, workplaces will be better placed to improve the safety of personnel who are directly impacted by sleep inertia and further prevent potential harm and loss of life.
